# Supplementary material for: MetaRibo-Seq measures translation in microbiomes
Source: Nat Commun. 2020 Jun 29;11:3268. doi: 10.1038/s41467-020-17081-z (PMC7324362; doi:10.1038/s41467-020-17081-z)
Supplement: Supplementary file 10 — Supplementary Data 7 [file 41467_2020_17081_MOESM10_ESM.zip › File2/Confidence_High_Taxonomy/162808_out.krona.html]

Javascript must be enabled to view this page.

members
magnitude
magnitudeUnassigned
count
unassigned
taxon
rank

162808\_out

164

2
164
superkingdom

phylum
164
976

164
class
200643

order
164
171549

1
family
1853231

283168
1
genus

species

SRS023526\_contig\_number\_34690
1
28118

family
159
2005525

1

SRS052078\_contig\_number\_2969
genus
375288
159

species

SRS019161\_contig\_number\_32621SRS019910\_contig\_number\_contig-100\_341.136523
2
387661

species
146

SRS011302\_contig\_number\_16672SRS011405\_contig\_number\_4900SRS013098\_contig\_number\_4142SRS013521\_contig\_number\_1367SRS013800\_contig\_number\_9882SRS013940\_contig\_number\_15619SRS014287\_contig\_number\_20221SRS014459\_contig\_number\_20849SRS014683\_contig\_number\_6417SRS014923\_contig\_number\_contig-100\_1357.230131SRS015065\_contig\_number\_37105SRS015264\_contig\_number\_16360SRS015431\_contig\_number\_26741SRS015579\_contig\_number\_15231SRS015663\_contig\_number\_6176SRS015694\_contig\_number\_10770SRS016018\_contig\_number\_6317SRS016056\_contig\_number\_1113SRS016335\_contig\_number\_48426SRS016381\_contig\_number\_2473SRS016989\_contig\_number\_5913SRS017859\_contig\_number\_3186SRS018049\_contig\_number\_790SRS018836\_contig\_number\_4982SRS018888\_contig\_number\_4074SRS018936\_contig\_number\_5662SRS019068\_contig\_number\_119674SRS019397\_contig\_number\_1255SRS019445\_contig\_number\_17490SRS019808\_contig\_number\_20382SRS020271\_contig\_number\_727SRS022137\_contig\_number\_1184SRS022524\_contig\_number\_16283SRS022609\_contig\_number\_6789SRS024009\_contig\_number\_contig-100\_361.78796SRS024435\_contig\_number\_34726SRS024492\_contig\_number\_19086SRS042628\_contig\_number\_38671SRS042966\_contig\_number\_15389SRS043667\_contig\_number\_4832SRS043841\_contig\_number\_8024SRS045004\_contig\_number\_27195SRS045244\_contig\_number\_3134SRS045645\_contig\_number\_31316SRS045826\_contig\_number\_18619SRS046068\_contig\_number\_1266SRS046369\_contig\_number\_4582SRS046717\_contig\_number\_9466SRS047433\_contig\_number\_19235SRS048981\_contig\_number\_2287SRS049446\_contig\_number\_14349SRS049773\_contig\_number\_37178SRS049959\_contig\_number\_1654SRS049959\_contig\_number\_49062SRS049995\_contig\_number\_15016SRS050299\_contig\_number\_10068SRS050422\_contig\_number\_32993SRS050520\_contig\_number\_911SRS050752\_contig\_number\_30404SRS050925\_contig\_number\_6286SRS050941\_contig\_number\_22675SRS051031\_contig\_number\_24231SRS051031\_contig\_number\_37062SRS051610\_contig\_number\_11527SRS052576\_contig\_number\_contig-100\_1611.17120SRS052697\_contig\_number\_2014SRS056519\_contig\_number\_10382SRS057480\_contig\_number\_contig-100\_2964.71066SRS058070\_contig\_number\_2185SRS058770\_contig\_number\_2491SRS062427\_contig\_number\_1117SRS062654\_contig\_number\_23962SRS063370\_contig\_number\_contig-100\_4577.73647SRS063985\_contig\_number\_13589SRS064232\_contig\_number\_3497SRS064276\_contig\_number\_40503SRS064757\_contig\_number\_7197SRS065176\_contig\_number\_17019SRS065397\_contig\_number\_10676SRS075773\_contig\_number\_contig-100\_481.230366SRS075821\_contig\_number\_15781SRS075984\_contig\_number\_6229SRS076804\_contig\_number\_4659SRS077086\_contig\_number\_6503SRS077589\_contig\_number\_3563SRS077641\_contig\_number\_16976SRS077849\_contig\_number\_11659SRS078176\_contig\_number\_639SRS078242\_contig\_number\_2073SRS078665\_contig\_number\_9443SRS097958\_contig\_number\_1750SRS098571\_contig\_number\_43671SRS098655\_contig\_number\_1720SRS098717\_contig\_number\_18203SRS101376\_contig\_number\_20834SRS101433\_contig\_number\_338SRS103987\_contig\_number\_1457SRS104036\_contig\_number\_8258SRS1041031\_contig\_number\_contig-100\_2538.152135SRS1041039\_contig\_number\_3636SRS1041091\_contig\_number\_20699SRS1041112\_contig\_number\_2611SRS1041116\_contig\_number\_2523SRS1041118\_contig\_number\_12249SRS1041134\_contig\_number\_6246SRS1041137\_contig\_number\_5621SRS1041139\_contig\_number\_7112SRS1041140\_contig\_number\_21184SRS1041141\_contig\_number\_3444SRS104327\_contig\_number\_859SRS104975\_contig\_number\_15009SRS105153\_contig\_number\_13590SRS1054716\_contig\_number\_11204SRS1055095\_contig\_number\_1011SRS140513\_contig\_number\_2173SRS142542\_contig\_number\_14287SRS142890\_contig\_number\_25107SRS142923\_contig\_number\_3642SRS143070\_contig\_number\_5716SRS143181\_contig\_number\_8539SRS143372\_contig\_number\_3036SRS143722\_contig\_number\_5884SRS143780\_contig\_number\_2107SRS144297\_contig\_number\_2860SRS144714\_contig\_number\_29749SRS146764\_contig\_number\_21986SRS146813\_contig\_number\_18996SRS147271\_contig\_number\_15012SRS147425\_contig\_number\_3636SRS147557\_contig\_number\_27401SRS147614\_contig\_number\_2612SRS148196\_contig\_number\_37807SRS148253\_contig\_number\_2120SRS148319\_contig\_number\_9709SRS148424\_contig\_number\_1905SRS148817\_contig\_number\_10596SRS149075\_contig\_number\_1797SRS149181\_contig\_number\_3569SRS893170\_contig\_number\_4783SRS893170\_contig\_number\_8082SRS893230\_contig\_number\_7569SRS893252\_contig\_number\_contig-100\_1401.115244SRS893358\_contig\_number\_10608SRS893378\_contig\_number\_4579SRS893382\_contig\_number\_1480SRS893383\_contig\_number\_21396
46503

1263094
10

SRS017859\_contig\_number\_2338SRS019397\_contig\_number\_9443SRS019968\_contig\_number\_12121SRS054059\_contig\_number\_3396SRS076876\_contig\_number\_920SRS1041136\_contig\_number\_22556SRS143148\_contig\_number\_451SRS145308\_contig\_number\_4961SRS893292\_contig\_number\_4011SRS893295\_contig\_number\_7348
species

4
family
815

816
genus
4

2292944
1

SRS050026\_contig\_number\_4916
species


SRS104912\_contig\_number\_9365
1
species
821

329854
species

SRS076976\_contig\_number\_15595
1

47678
species
1

SRS024132\_contig\_number\_21791
